# Remodelling of the erythrocyte cytoskeleton during *P. falciparum* merozoite invasion

**DOI:** 10.1186/1475-2875-13-S1-P96

**Published:** 2014-09-22

**Authors:** Elizabeth Zuccala, Timothy Satchwell, Fiona Angrisano, Danushka Marapana, Yan-Hong Tan, Ashley Toye, Jake Baum

**Affiliations:** 1 Division of Infection & Immunity, Walter & Eliza Hall Institute of Medical Research, Parkville, Victoria, Australia; 2 Department of Medical Biology, University of Melbourne, Parkville, Victoria, Australia; 3 Department of Life Sciences, Imperial College London, South Kensington, London, UK; 4 School of Biochemistry, University of Bristol, Bristol, UK; 5 Bristol Institute for Transfusion Sciences, NHS Blood & Transplant, Bristol, UK

## Background

Red blood cells are remarkably resilient, flexible and dynamic structures. These properties are required for their passage through small capillaries and are imparted by the cytoskeleton, a network of proteins that underlies and links to the cell membrane. To successfully invade, the blood stage malaria parasite must induce drastic changes to the structure of the target erythrocyte, including the formation of a tight junction and a new cellular compartment, the parasitophorous vacuole. These key modifications involve the infolding of the red blood cell membrane, membrane fusion and fission events and the secretion of parasite proteins into the host. While detailed cellular descriptions of merozoite invasion have been achieved over the past few decades, comparatively little is known about the molecular basis of how the host cell responds to parasite entry. It has long been suggested that erythrocyte ATP and a kinase are required for successful invasion, while more recently a role for a host-cell actin polymerisation in invasion has been posited. We hypothesize that to invade, merozoites interface with endogenous erythrocyte pathways that regulate membrane and cytoskeletal remodelling, and here present cellular and molecular data on the host factors involved in facilitating merozoite invasion.

## Materials and methods

In order capture invasion events we isolated free *Plasmodium falciparum* merozoites. Using this method, invasion inhibition was assayed by flow cytometry, fluorescence imaging of invading parasites was performed and quantitative phosphor-proteomics of invasion samples was achieved using TMT-labeling.

## Results

To investigate the role of an active host cell in invasion, we used a non-hydrolysable ATP analogue and ATP depletion of erythrocytes. Treated erythrocytes showed a reduced capacity to support merozoite invasion. Using a combination of high definition imaging and inhibitors of actin dynamics, we found erythrocyte actin does not appear to polymerise around the invading parasite, and its turnover is likely not required for successful entry. A role for host cell kinase activity was explored using inhibitors and through quantitative phosphoproteomics, which revealed post-translational modifications to the erythrocyte cytoskeleton in response to merozoites.

## Conclusions

We explored the possibility that the erythrocyte is not a passive bystander during invasion, but rather actively responds to merozoite contact and potentially contributes to parasite entry through the remodelling of its cytoskeleton. By shedding light on the host-cell contribution to invasion we hope to uncover novel chemotherapeutic targets to stop invasion and hence prevent or treat malaria disease.

